# The impact of single versus mixed schistosome species infections on liver, spleen and bladder morbidity within Malian children pre- and post-praziquantel treatment

**DOI:** 10.1186/1471-2334-10-227

**Published:** 2010-07-29

**Authors:** Artemis Koukounari, Christl A Donnelly, Moussa Sacko, Adama D Keita, Aly Landouré, Robert Dembelé, Elisa Bosqué-Oliva, Albis F Gabrielli, Anouk Gouvras, Mamadou Traoré, Alan Fenwick, Joanne P Webster

**Affiliations:** 1 Schistosomiasis Control Initiative, Department of Infectious Disease Epidemiology, Faculty of Medicine, Imperial College London, UK; 2 MRC Centre for Outbreak Analysis and Modelling, Department of Infectious Disease Epidemiology, Imperial College London, London, UK; 3 Institut National de Recherche en Santé Publique, Ministère de la Santé, Bamako, Mali; 4 Service de la Radiologie, Hôpital National du Point G, Bamako, Mali; 5 Programme National de Lutte contre la Schistosomiase et les Géohelminthiases, Direction Nationale de la Santé, Ministère de la Santé, Bamako, Mali; 6 Department of Control of Neglected Tropical Diseases, World Health Organization, CH-1211 Geneva 27, Switzerland

## Abstract

**Background:**

In the developing world co-infections and polyparasitism within humans appear to be the rule rather than the exception, be it any combination of inter-specific and/or inter- and intra-Genera mixed infections. Mixed infections might generate synergistic or antagonistic interactions and thereby clinically affect individuals and/or impact parasite epidemiology.

**Methods:**

The current study uniquely assesses both *Schistosoma mansoni*- and *Schistosoma haematobium*-related morbidity of the liver and the bladder as assessed by ultrasound as well as spleen and liver morbidity through clinical exams. The impact of praziquantel (PZQ) treatment on such potential inter-specific schistosome interactions and resulting morbidity using uniquely detailed longitudinal data (pre- and one year post-PZQ treatment) arising from the National Schistosomiasis Control Program in three areas of Mali: Ségou, Koulikoro and Bamako, is also evaluated. At baseline, data were collected from up to 2196 children (aged 7-14 years), 844 of which were infected with *S. haematobium *only, 124 with *S. mansoni *only and 477 with both. Follow-up data were collected from up to 1265 children.

**Results:**

Results suggested lower liver morbidity in mixed compared to single *S. mansoni *infections and higher bladder morbidity in mixed compared to single *S. haematobium *infections. Single *S. haematobium *or *S. mansoni *infections were also associated with liver and spleen morbidity whilst only single *S. haematobium *infections were associated with bladder morbidity in these children (light *S. haematobium *infection OR: 4.3, p < 0.001 and heavy *S. haematobium *infection OR: 19, p < 0.001). PZQ treatment contributed to the regression of some of the forms of such morbidities.

**Conclusions:**

Whilst the precise biological mechanisms for these observations remain to be ascertained, the results illustrate the importance of considering mixed species infections in any analyses of parasite-induced morbidity, including that for the proposed Disability Adjusted Life Years (DALYs) revised estimates of schistosomiasis morbidity.

## Background

In the developing world co-infections and polyparasitism within humans appear to be the rule rather than the exception. New integrated control programmes acknowledge this, with for instance combined mass drug administration (MDA) for schistosomiasis, lymphatic filariasis, onchocerciasis, trachoma and soil-transmitted helminths being initiated throughout parts of sub-Saharan Africa [1]. However, within-Genera polyparasitism must also be fully acknowledged. Moreover, synergistic or antagonistic interactions resulting from such co-infections may be predicted to clinically affect individuals and/or impact parasite epidemiology [2].

Schistosomiasis is a chronic and debilitating disease which affects millions of people, particularly the rural poor in the developing world. Of some 600 million people at risk, an estimated 200 million are infected, more than half of which are symptomatic and at least 20 million exhibit severe disease manifestations [3]. Schistosomes, the causative agents, are parasitic bloodflukes (Phylum: Platyhelminth; Class: Trematoda) with indirectly transmitted life-cycles involving obligatory alternation of generations between sexual reproduction in a mammalian host and asexual reproduction within a molluscan (freshwater snail) host. The clinical manifestations of schistosomiasis are classically associated with the species-specific ovipositioning (egg-laying) sites; the mesenteric venous systems for *Schistosoma mansoni *(prevalent in sub-Saharan Africa and South America) leading to chronic hepatic and intestinal fibrosis and the vesical venous plexus of the urogenital system for *Schistosoma haematobium *(prevalent in sub-Saharan Africa) associated with ureteral and bladder fibrosis, calcification of the urinary tract and bladder cancer [4].

Co-infections between *S. mansoni *and *S. haematobium *have been reported in an increasing number of foci across Africa [5-9]. Furthermore, studies in Cameroon and Senegal have revealed that in areas of overlap, *S. mansoni*-shaped eggs may be excreted in urine as opposed to the usual faecal route [10]. Studies on schistosomiasis-associated morbidity tend to, nevertheless, always consider single species infections individually [3]. In addition to affecting the prevalence and intensity of human infections, inter-specific parasite interactions during mixed species infections may, however, also be predicted to impact host morbidity directly. In one of the few studies to consider this, Cunin et al. (2003) in Cameroon, for example, reported an apparent lowering of *S. mansoni-*induced morbidity (hepatomegaly and splenomegaly morbidity, as determined through clinical palpation) in mixed infections with *S. haematobium *relative to that observed for *S. mansoni *single infections [6]. The authors suggested that this lowering effect on liver morbidity could be due to *S. haematobium *males mating with *S. mansoni *females (which cannot successfully hybridize) and the subsequent eggs produced from such couplings passing to the urinary oviposition site, thereby reducing the amount of classical *S. mansoni*-induced morbidity. Laboratory rodent model studies have confirmed these observations [11].

Preventive mass drug administration (MDA) interventions, as are recently being employed across parts of sub-Saharan Africa [12,13], may be further predicted to have an impact on such complex inter-specific interactions, especially if the selection pressure differs between the parasite species, as may be plausible for *S. mansoni *and *S. haematobium *[14]. For example, *S. mansoni *and *S. haematobium *differ in their generation times/length of time to maturation, and as juvenile schistosomes are not susceptible to PZQ, this could potentially lead to differences between these two species in terms of their responses to MDA within individual hosts. Such differences could also subsequently affect the order of establishment of the parasites within their human hosts (a factor known to be very important in determining the outcome of inter-specific competition and subsequent parasite-induced host morbidity [15,9]).

However, despite the theoretical and applied interest of mixed schistosome species interactions on human host morbidity under differing selective pressures, few studies appear to have focused upon this. Indeed, to the authors knowledge, no studies have yet examined the potential consequence of *S. haematobium*-associated bladder pathology in mixed species infections, nor the potential impact of MDA on hepatic and urinary-associated morbidity of mixed schistosome species infections.

The aim of the current study was to test if the hypothesis that liver morbidity in mixed schistosome species infections is lower relative to that observed for *S. mansoni *single infections [6], can be generalized in different environmental settings within sub-Saharan Africa such as Mali. Furthermore, while previous studies were restricted to clinical examination and palpation of hepatomegaly and splenomegaly, the present study also incorporates detailed ultrasonography (US) to determine schistosome-specific morbidity in the liver and bladder. Finally, the current study also evaluates the impact of PZQ treatment on such potential inter-specific schistosome interactions and resulting morbidity using uniquely detailed longitudinal data (pre- and one year post-praziquantel (PZQ) treatment) arising from the National Schistosomiasis Control Program in three areas of Mali: Ségou, Koulikoro and Bamako. The results obtained should have theoretical and applied implications regarding inter-specific schistosome interactions and human morbidity.

## Methods

### Study design

The schools included in these surveys were randomly selected from all schools known *a priori *to be places where schistosomiasis is highly endemic: Ségou, Bamako and Koulikoro. Details concerning sample-size calculations and cohort design have been described elsewhere [16]. We would like to acknowledge that this study describes data from an ongoing MDA control programme and not a double-blind placebo randomized clinical trial study.

Initially 2196 children (aged 7 to 14 years) from 29 schools were recruited with US and parasitological data on both schistosome species infections. Of these, 844 children were infected with *S. haematobium *only, 124 with *S. mansoni *only and 477 with both. Clinical examinations (i.e. liver and spleen palpations) were performed on 2128 of these children, as part of the baseline survey (of these, 821 children were infected with *S. haematobium *only, 117 with *S. mansoni *only and 463 with both).

Only 23 of the 29 schools surveyed at baseline were visited at the second survey, due to financial and time constraints. All subsequent analyses here of the follow-up (second survey) data exclude baseline data from the 6 schools that were not followed up. Follow-up data were collected from 1265 children from 23 schools (76% of the 1670 children recruited from these schools at baseline) but only 853 children had complete clinical, US and parasitological data on both schistosomiasis infections. Follow-up rates following the first survey were much higher for children under 13 years of age, as is common in such sub-Saharan regions where primary school children leave for reasons such as work or early marriage [17].

Baseline surveys took place in the Ségou area in March and April of 2004 while the follow-up surveys were performed just over one year later (i.e. in May 2005). By the second survey children in the schools of this area had received two MDA treatments with PZQ: a) one just after baseline data collection (in March-April 2004) and b) another during preventive chemotherapy intervention (in February 2005). In Bamako baseline surveys took place in July 2004 while the majority of the follow-up surveys were performed almost two years later (i.e. in April-May 2006) with the remaining three schools in this area to be visited in October 2006. A single round of MDA took place in 2005 (i.e. in May, June and November). Finally baseline surveys took place in Koulikoro during June-August 2004 while the follow-up surveys took place almost two years later (i.e. in May 2006). The children would have received one PZQ treatment during the MDA in August 2005.

### Clinical, ultrasound and parasitological examination

Clinical examination was performed on each child by experienced clinical nurses who were blind to parasitological results. The following measurements were recorded: the excursion in centimeters of the spleen below the rib cage in the left mid-clavicular line (MCL) and left mid-axillary line (MAL); liver tenderness; the excursion in centimeters of the left liver lobe beneath the sternum in the mid-sternal line (MSL); the excursion of the right liver lobe beneath the rib cage in the right MCL. The consistency of the liver and spleen were also graded as follows: not palpable, soft, firm and hard. The presence of physical abnormalities such as ascites or other abdominal swelling, umbilical collaterals and scars were recorded as being present or absent, and finally, the presence of liver tenderness or febrility were noted [18].

Ultrasound (US) examination was performed with a portable ultrasonography device (SSD-500; Aloca, Tokyo, Japan). For parasitologic examinations, a filtration method was used to determine *S. haematobium *infection intensity, whilst the Kato-Katz (KK) technique was employed to define *S. mansoni *infection intensity. Further details have been described elsewhere for both US and KK [16].

### Ethics statement

Ethical approval was obtained from the St Mary's Hospital Local Ethics Research Committee, R&D office (part of the Imperial College, London Research Ethics Committee (ICREC)) in combination with the ongoing Schistosomiasis Control Initiative (SCI) activities, in areas where SCI is physically based. Within Mali, all aspects of Monitoring and Evaluation were carried out in the framework of the disease control activities implemented and approved by the Ministry of Health (MOH) and adopted by regional and local administrative and health authorities. The communities of the selected villages were informed about the objectives, the methodology of the study and the advantages. A meeting was organized with the population and verbal community consent was obtained for each selected village. Where the study was performed within the schools, verbal consent was also obtained from school teacher's directors and teachers, prior to the recruitment of the children. Within Mali, for cultural reasons, obtaining verbal consent from the community leaders/heads of villages (via community forum), and teachers' directors, is the most accepted procedure, and hence the practice we followed here.

The results of the different diagnostic procedures performed on children were briefly explained to the children themselves. If any pathology was discovered, this was reported to the parents or guardians, or, if this was not possible, carefully explained to the teachers for them to brief the children's parents or guardians. The health officer responsible for the nearest medical post, who was usually present on spot at the time of the examinations, was also informed about the pathology of the children and asked to take care of them, according to the practices and procedures of the national health system of Mali.

### Statistical Analyses

Baseline (pre-MDA) data were used to investigate whether or not there was an association between schistosomiasis infection status and pathological manifestations as determined by US examinations for liver and bladder morbidity as well as by clinical examinations for liver and spleen morbidities. Univariate analyses were performed using chi-square tests for associations between schistosomiasis infection status and the various pathological manifestations.

In order to study simultaneously the impact of several covariates on the pathological manifestations at baseline, logistic regression models with random effects at the school level (i.e. multilevel logistic regressions) were fitted using the PROC NLMIXED command in SAS version 9.1 (SAS Institute Inc., Cary, NC). Schistosomiasis infections were classified on the basis of intensities using WHO standards (see S1a in Additional file 1), and these intensity classes were included as covariates. The assumption of linear effects of age was tested through comparison with models including categorical effects for each year of age. The assumption of linear effects of infection intensity classes was tested using the Akaike information criterion (AIC) to compare models assuming linear effects (with and without an interaction of those linear effects) with models fitting separate effects for each combination of *S. mansoni *and *S. haematobium *intensity classes. The intensity classes for *S. haematobium *infection were assigned 0 for 'not infected' (0e/10 ml), 1 for 'light' (< 50e/10 ml) and 2 for 'heavy' (> 50e/10 ml) while the intensity classes for *S. mansoni *infection were assigned 0 for 'not infected' (0 epg), 1 for 'light' (1-99 epg), 2 for 'medium' (100-399 epg) and 3 for 'heavy' (> 400 epg).

Differences in baseline characteristics were tested between children successfully and unsuccessfully followed up by univariate analysis using a Wilcoxon 2-sample test for means and a chi-square test or Fisher's exact test if there were small values for proportions. The data were further analyzed excluding data from 13- and 14-year-old children because children in these age groups would be more likely to have left school during interval between baseline and follow-up assessments.

Statistical analysis for the effect of MDA (i.e. post PZQ treatment, one year follow-up) was restricted to children with complete records on all outcomes of interest here (i.e. parasitological, US and clinical exam data) from both baseline and follow-up surveys. Differences in baseline and separately follow-up health characteristics of children between pairs of the three surveyed areas of the country were performed using two-proportion z-tests of unequal variances. Such comparisons were performed as different treatment strategies had been followed between these regions.

Changes of the health characteristics of children between the two time points of the study in relation to their baseline schistosomiasis infection status, adjusting at the same time for age and sex, were analyzed using generalized logit multinomial models for all three areas and for each morbidity measure using the PROC LOGISTIC command in SAS version 9.1 (SAS Institute Inc., Cary, NC). In these models schistosome infection status was characterized as simply infected or not infected, rather than by the intensity of infection, based on model fits with prevalence and linear effects of intensity (see S3 in Additional File 1 for further details).

Finally, in these models the response variable was a new categorical variable with four levels indicating the following: a) no pathological manifestation of each morbidity measure at both time points, b) pathological manifestation of each morbidity measure at baseline but cured at final point of the study, c) no pathological manifestation of each morbidity measure at baseline and pathological manifestation of each morbidity measure at final point of the study, or d) pathological manifestation of each morbidity measure at both time points.

## Results

The ages of children were found to be approximately matched between infection status groups. The mean ages were: for the uninfected children 10.13 years old; for the children with single *S. haematobium *infection 10.52 years old; for the children with single *S. mansoni *infection 10.96 years old; for those children who were co-infected with both schistosomes species the mean age was 10.64 years old.

### Univariate analysis for the association between schistosomiasis infection status and pathological manifestations at baseline

**Figure 1:**
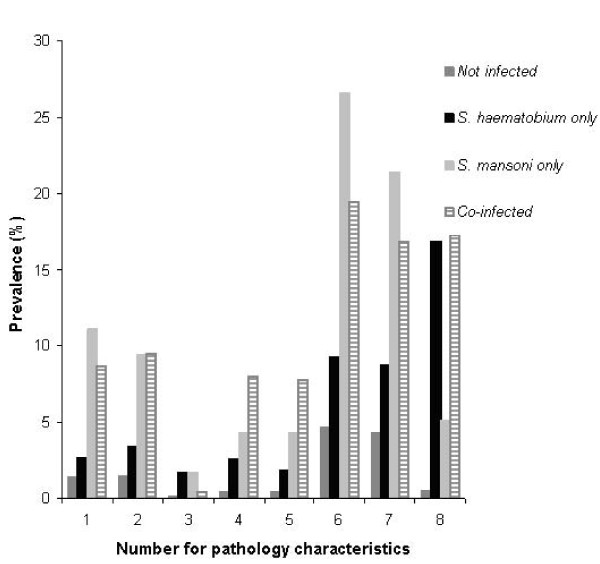
**Univariate analysis for association between single and mixed schistosomiasis infections with liver, bladder and spleen pathology as assessed by US and clinical examination at baseline (n = 2128 children)**. Numerical coding for pathology characteristics in the horizontal axis of Figure 1 represent the following: 1: Abnormal liver image patterns as assessed by US examination; 2: Portal Hypertension (as determined by positive PVD scores from US examination); 3: Hepatomegaly as determined by positive PSL scores from US examination; 4: Hepatomegaly as determined by MSL>2 cm from clinical examination; 5: Hepatomegaly as determined by MCL>2 cm from clinical examination; 6: Splenomegaly as determined by MCL>2 cm from clinical examination; 7: Splenomegaly as determined by MAL>2 cm from clinical examination; 8: Bladder pathology (as determined by positive global scores from US examination). US, ultrasound; PVD, portal vein diameters: PSL, parasternal line; MSL, mid-sternal line; MCL, mid-clavicluar line; MAL, mid-axillary line.

Figure 1 presents baseline prevalence levels of liver or spleen morbidity as determined by US or clinical examinations and bladder morbidity as determined by US examination for those with single and both schistosome species infections as well as those who were uninfected. Chi-square tests revealed that the prevalence of liver and spleen morbidity (pathology characteristics 1 to 7 in Figure 1) did not differ significantly in children with *S. mansoni *infection only compared with co-infected children. Similarly, the bladder morbidity (pathology characteristic 8, in Figure 1) did not differ significantly in children with *S. haematobium *infection only compared with co-infected children.

However, the prevalence levels of liver morbidity in the forms of portal hypertension and hepatomegaly, as assessed both by US, were significantly higher among children infected with *S. haematobium *only than among uninfected children (p = 0.017 & p = 0.002 respectively for pathology characteristics 2 and 3 in Figure 1). Similarly, clinical examination revealed significantly higher prevalence levels of liver morbidity among children infected with *S. haematobium *only than among uninfected children (p = 0.001 and p = 0.010 respectively for pathology characteristics with numbers 4 and 5 in Figure 1).

Clinical examination revealed significantly higher prevalence levels of spleen morbidity among children infected with *S. haematobium *only than among uninfected children (p < 0.001 for both pathology characteristics 6 and 7 in Figure 1). Finally, the prevalence level of bladder morbidity was significantly higher among children infected with *S. haematobium *only than among uninfected children (p < 0.001 for pathology characteristic 8 in Figure 1).

### Multivariate analyses for the association between the intensity of schistosomiasis infection status and pathological manifestations at baseline

Models with linear effects of intensities yielded better fit through AIC comparisons and these are the results that are presented here (see S1b in Additional File 1). The adjusted odds ratios (ORs) for gender and age from multilevel multivariate logistic regression models for the liver pathology outcomes, as assessed by US examination, are shown in Table 1. Estimates for the risk of having an abnormal liver image pattern indicated that children infected with *S. mansoni *only (light, medium and heavy intensities) were significantly more likely to have an abnormal liver image pattern compared to uninfected children (OR: 1.6, p = 0.008; OR: 2.5, p = 0.008 and OR:3.9, p = 0.008 respectively), while the data indicated that co-infected children were at lower risk of liver morbidity, in the form of an abnormal liver image pattern, than children with single *S. mansoni *species infections of the same intensity. Furthermore, there were no significantly increased risks of liver morbidity, in the form of an abnormal liver image pattern, among the co-infected children than among the uninfected children. Children infected with *S. haematobium *only (both light and heavy intensities) were significantly less likely to have an abnormal liver image pattern compared to uninfected children (OR: 0.5, p = 0.003 and OR: 0.3, p = 0.003 respectively).

**Table 1:** Multivariate logistic regressions for liver pathology as assessed by US examination at baseline (n = 2128)

Estimates from multilevel logistic regressions for liver pathology as assessed by US				
	Adjusted ORs for gender and age (95% CI) and p-values for the risk of having abnormal liver image pattern			
*Type of schistosomiasis infection intensity related variables*	*For S. mansoni*			
***For S. haematobium***	**none**	**light**	**medium**	**heavy**
none	1	1.569	2.463	3.866
		(1.123 to 2.194)	(1.261 to 4.812)	(1.416 to 10.556)
		p = 0.008	p = 0.008	p = 0.008
Light	0.526	0.825	1.295	2.033
	(0.343 to 0.807)	(0.520 to 1.309)	(0.654 to 2.565)	(0.769 to 5.374)
	p = 0.003	p = 0.415	p = 0.458	p = 0.153
Heavy	0.277	0.434	0.681	1.069
	(0.117 to 0.651)	(0.190 to 0.990)	(0.271 to 1.715)	(0.350 to 3.267)
	p = 0.003	p = 0.047	p = 0.415	p = 0.907
	**Adjusted ORs for gender and age (95% CI) and p-values for the risk of having portal hypertension as assessed by positive PVD scores**			
	***For S. mansoni***			
***For S. haematobium***	none	light	medium	heavy
None	1	0.882	0.778	0.686
		(0.655 to 1.187)	(0.429 to 1.410)	(0.281 to 1.674)
		p = 0.408	p = 0.408	p = 0.408
Light	1.013	0.893	0.788	0.695
	(0.698 to 1.470)	(0.582 to 1.372)	(0.417 to 1.489)	(0.284 to 1.703)
	p = 0.946	p = 0.606	p = 0.463	p = 0.426
Heavy	1.026	0.905	0.798	0.704
	(0.487 to 2.161)	(0.429 to 1.908)	(0.339 to 1.881)	(0.248 to 2.000)
	p = 0.946	p = 0.793	p = 0.606	p = 0.510
	**Adjusted ORs for gender and age (95% CI) and p-values for the risk of having of hepatomegaly as assessed by positive PSL scores**			
	***For S. mansoni***			
***For S. haematobium***	none	light	medium	heavy
None	1	4.583	21.006	96.274
		(1.517 to 13.844)	(2.301 to 191.788)	(3.490 to 2656.018)
		p = 0.007	p = 0.007	p = 0.007
Light	3.409	4.705	6.494	8.964
	(1.273 to 9.125)	(1.201 to 18.425)	(0.777 to 54.292	(0.453 to 177.253)
	p = 0.015	p = 0.026	p = 0.084	p = 0.150
Heavy	11.619	4.83	2.008	0.835
	(1.621 to 83.261)	(0.536 to 43.480)	(0.077 to 52.677)	(0.008 to 85.355)
	p = 0.015	p = 0.160	p = 0.676	p = 0.939

The categories for the intensity of *S. haematobium *infection were defined according to WHO guidelines as: none: 0 e/10 ml; light: < 50 e/10 ml and heavy: ≥ 50 e 10 ml.

The categories for the intensity of *S. mansoni *infection were defined according to WHO guidelines as: none: 0 epg; light: 1-99 epg; moderate: 100-399 epg; and heavy: ≥ 400 epg

e/10 ml, eggs/10 milliliters & epg, eggs per gram

These categorizations for both schistosomes species apply for all tables presented beyond this point.

ORs, Odds Ratios; 95% CIs, 95% Confidence Intervals

As regards the risk of having portal hypertension, the multilevel multivariate logistic regression model (Table 1) did not yield any significant associations with any of the intensities of the single or mixed schistosome species.

Regarding the risk of having hepatomegaly as assessed by positive PSL scores, children with single infections (*S. mansoni *only or *S. haematobium *only) were more likely to have this marker of liver morbidity than uninfected children (with children with medium and heavy intensity *S. mansoni *infections only being at particularly high risk: OR: 21, p = 0.007 and OR = 96, p = 0.007, respectively). The data indicated that children with medium and high intensity *S. mansoni *infections, but no *S. haematobium *infection, were at substantially higher risk of liver morbidity of this form than co-infected children with medium and high intensity *S. mansoni *infections. Furthermore, there were no significantly increased risks of liver morbidity, as assessed by positive PSL scores, among the co-infected children with medium and high intensity *S. mansoni *infections than among the uninfected children.

The risk of having hepatomegaly as assessed by MSL>2 cm (Table 2) appeared to depend only upon the intensity of the *S. mansoni *infection (risk was lowest with no infection and increased with increased *S. mansoni *intensity), with no effect of any *S. haematobium *infection. Similarly, the risk of having hepatomegaly as assessed by MCL>2 cm (Table 2) appeared to depend only upon the intensity of the *S. mansoni *infection (risk was lowest with no infection and increased with increased *S. mansoni *intensity), with no effect of any *S. haematobium *infection.

**Table 2:** Multivariate logistic regressions for liver and spleen pathology as assessed by clinical examination at baseline (n = 2128)

Estimates from multilevel logistic regressions				
	Adjusted ORs for gender and age (95% CI) and p-values for the risk of having hepatomegaly as assessed by MSL >2 cm			
*Type of schistosomiasis infection intensity related variables*	*For S. mansoni*			
*For S. haematobium*	none	light	medium	heavy
None	1	1.431	2.049	2.932
		(1.008 to 2.033)	(1.016 to 4.132)	(1.024 to 8.399)
		p = 0.045	p = 0.045	p = 0.045
Light	0.995	1.425	2.039	2.919
	(0.614 to 1.612)	(0.839 to 2.419)	(0.956 to 4.349)	(1.016 to 8.383)
	p = 0.985	p = 0.190	p = 0.065	p = 0.047
Heavy	0.991	1.418	2.03	2.905
	(0.377 to 2.600)	(0.548 to 3.667)	(0.704 to 5.850)	(0.825 to 10.230)
	p = 0.985	p = 0.471	p = 0.190	p = 0.097
	**Adjusted ORs for gender and age (95% CI) and p-values for the risk of having hepatomegaly as assessed by MCL>2 cm**			
	***For S. mansoni***			
***For S. haematobium***	none	light	medium	heavy
None	1	1.446	2.092	3.025
		(1.006 to 2.078)	(1.013 to 4.320)	(1.019 to 8.979)
		p = 0.046	p = 0.046	p = 0.046
Light	1.057	1.529	2.211	3.198
	(0.652 to 1.713)	(0.895 to 2.611)	(1.017 to 4.805)	(1.078 to 9.484)
	p = 0.822	p = 0.120	p = 0.045	p = 0.036
Heavy	1.117	1.616	2.337	3.38
	(0.425 to 2.934)	(0.623 to 4.189)	(0.801 to 6.815)	(0.937 to 12.196)
	p = 0.822	p = 0.323	p = 0.120	p = 0.063
	**Adjusted ORs for gender and age (95% CI) and p-values for the risk of having splenomegaly as assessed by MCL>2 cm**			
	***For S. mansoni***			
***For S. haematobium***	none	light	medium	heavy
None	1	1.068	1.142	1.22
		(0.864 to 1.321)	(0.747 to 1.745)	(0.645 to 2.306)
		p = 0.541	p = 0.541	p = 0.541
Light	0.768	0.82	0.876	0.936
	(0.594 to 0.992)	(0.607 to 1.108)	(0.557 to 1.378)	(0.494 to 1.776)
	p = 0.044	p = 0.197	p = 0.568	p = 0.840
Heavy	0.589	0.629	0.673	0.719
	(0.352 to 0.985)	(0.375 to 1.057)	(0.368 to 1.228)	(0.343 to 1.505)
	p = 0.044	p = 0.080	p = 0.197	p = 0.381
	**Adjusted ORs for gender and age (95% CI) and p-values for the risk of having splenomegaly as assessed by MAL>2 cm**			
	***For S. mansoni***			
***For S. haematobium***	none	light	medium	heavy
None	1	1.099	1.207	1.326
		(0.879 to 1.373)	(0.773 to 1.886)	(0.679 to 2.591)
		p = 0.408	p = 0.408	p = 0.408
Light	0.754	0.829	0.911	1.001
	(0.576 to 0.988)	(0.604 to 1.139)	(0.565 to 1.468)	(0.510 to 1.965)
	p = 0.041	p = 0.247	p = 0.701	p = 0.998
heavy	0.569	0.625	0.687	0.755
	(0.332 to 0.977)	(0.362 to 1.079)	(0.364 to 1.296)	(0.346 to 1.646)
	p = 0.041	p = 0.092	p = 0.247	p = 0.480

ORs, Odds Ratios; 95% CIs, 95% Confidence Intervals

The risk of having splenomegaly as assessed once by MCL>2 cm and then by MAL>2 cm did not depend on the presence, or intensity of single or mixed *S. mansoni *infection but it was slightly reduced in the presence of single *S. haematobium *infection (Table 2).

The risk of bladder pathology as assessed by US examination (Table 3) did not depend on the presence, or intensity of single *S. mansoni *infection but was much increased in the presence of single *S. haematobium *infection (light *S. haematobium *infection OR: 4.3, p < 0.001 and heavy *S. haematobium *infection OR: 19, p < 0.001 for children uninfected with *S. mansoni *in both cohorts) and even further in the presence of mixed infections.

**Table 3:** Multivariate logistic regression of bladder pathology as assessed by US at baseline (n = 2128)

Estimates from multilevel logistic regressions				
	Adjusted ORs for gender and age (95% CI) and p-values for the risk of having bladder pathology as assessed by positive global scores			
*Type of schistosomiasis infectionintensity related variables*	*For S. mansoni*			
*For S. haematobium*	none	light	medium	heavy
None	1	1.166	1.36	1.587
		(0.923 to 1.473)	(0.853 to 2.170)	(0.787 to 3.198)
		p = 0.197	p = 0.197	p = 0.197
Light	4.347	5.07	5.914	6.898
	(3.232 to 5.847)	(3.560 to 7.220)	(3.513 to 9.955)	(3.339 to 14.251)
	p < 0.001	p < 0.001	p < 0.001	p < 0.001
Heavy	18.897	22.041	25.708	29.985
	(10.446 to 34.186)	(11.986 to 40.531)	(12.677 to 52.134)	(12.702 to 70.783)
	p < 0.001	p < 0.001	p < 0.001	p < 0.001

ORs, Odds Ratios; 95% CIs, 95% Confidence Intervals

### Drop out rates at follow-up

There were significant differences in the ages of the 853 children successfully followed up and the 817 children who dropped out during the follow-up (p < 0.001); children who dropped out were older on average than children who were successfully followed up (i.e. 11.25 years versus 9.62 years). There were also significant differences between these two groups of children with reference to the prevalence of abnormal liver image patterns (p = 0.024) and the bladder pathology (p = 0.010), as determined by US examination, as well as the prevalence of hepatomegaly and splenomegaly as determined by clinical examination (p = 0.020 and p = 0.003 respectively). These indicators of morbidity were significantly lower at baseline among children that dropped out than in children who were successfully followed up. For all other pathological manifestations considered, there were no significant differences between these two groups of children: those who dropped out and those who were successfully followed up.

Excluding data from children aged 13 and 14 years at baseline, the only significant follow-up differences were that the prevalences of mixed schistosome species infections and bladder pathology were significantly lower in those children who dropped out compared to those who were successfully followed up. The observation that most features examined were similar in children that were successfully followed up and children who dropped out during the follow-up suggested that results obtained, after appropriate adjustment for age, were largely representative of the population from which the sample had been selected (see S2 in Additional File 1 for further details).

### Impact of MDA

Both at baseline and at follow-up the heaviest intensities of *S. haematobium *infection were observed in Ségou while the heaviest intensities of *S. mansoni *infection were observed in Koulikoro (Table 4). However, in all three areas and for both schistosome species, there were observed significant decreases from baseline to follow-up in the classes of heavy intensities with the exception of Bamako area and the heavy intensity of *S. mansoni *infection. It should be noted though that the latter was very low at baseline and thus even if there was a significant drop post treatment, it was unlikely to have been detected with this small sample size. Co-infections also decreased significantly in all three areas at follow-up relative to baseline.

**Table 4:** Health characteristics of schoolchildren successfully retraced (i.e. pre and post treatment-n = 853)

	Baseline			Post-treatment		
	*Bamako*	*Koulikoro*	*Ségou*	*Bamako*	*Koulikoro*	*Ségou*
	*(n = 273)*	*(n = 153)*	*(n = 427)*	*(n = 273)*	*(n = 153)*	*(n = 427)*
**Parasitology**						
% Uninfected	45.05 (< 0.001)a	14.38 (< 0.001)b	8.90 (< 0.001)c	64.10 (< 0.001)d **	32.68 (0.010)e **	40.05 (< 0.001)f **
% Infected with *S. haematobium*	43.96 (< 0.001)a	3.92 (< 0.001)b	57.38 (< 0.001) c	26.37 (< 0.001)d **	5.23 (< 0.001)e	35.36 (0.011) f **
% Infected with *S. mansoni*	1.10 (< 0.001)a	24.84 (< 0.001)b	4.22 (0.007)c	4.03 (< 0.001)d **	40.52 (< 0.001)e **	14.05 (< 0.001)f **
% Co-infected	9.89 (< 0.001)a	56.86 (< 0.001)b	29.51 (< 0.001)c	5.49 (< 0.001)d **	21.57 (0.002)e **	10.54 (0.013) f **
% Heavy *S. haematobium *infections	13.19 (0.040) a	7.19 (< 0.001) b	39.58 (< 0.001)	2.20 (0.161) c **	0.65 (< 0.001) e **	7.73 (< 0.001) f **
% Heavy *S. mansoni *infections	0.73 (< 0.001)	22.88 (0.005)	12.41 (< 0.001)	0.00 (< 0.001)	10.46 (0.003) **	2.81 (< 0.001) **
**Liver Pathology as assessed by ultrasound (US) examination**						
% with abnormal liver image patterns	0.37 (0.316)a	0.00 (< 0.001)b	12.18 (< 0.001)c	0.37 (0.316)d	0.00 (< 0.001)e	12.88 (< 0.001)f
% with portal hypertension (as determined by positive PVD scores)	0.73 (0.585)a	1.31 (< 0.001)b	9.60 (< 0.001)c	5.49 (0.013)d **	13.07 (0.076)e **	7.73 (0.237)f
% with hepatomegaly (as determined by positive PSL scores from US)	0.37 (0.701)a	0.65 (0.085)b	2.34 (0.085)c	0.00 (0.316)d	0.65 (0.545)e	0.23 (0.317)f **
**Liver + spleen pathology as assessed by clinical examination**						
% with hepatomegaly as assessed by MSL>2 cm	0.00 (0.316)a	0.65 (< 0.001)b	9.84 (< 0.001)c	0.00 (0.080)d	1.96 (0.291)e	0.70 (0.082)f **
% with hepatomegaly as assessed by MCL>2 cm	0.00 (0.316)a	0.65 (< 0.001)b	8.67 (< 0.001)c	0.00 (0.080)d	1.96 (0.291)e	0.70 (0.082)f **
% with splenomegaly as assessed by MCL>2 cm	0.73 (< 0.001)a	12.42 (< 0.001)b	27.17 (< 0.001)c	0.00 (0.023)d	3.27 (0.009)e **	8.43 (< 0.001)f **
% with splenomegaly as assessed by MAL>2 cm	0.73 (< 0.001)a	8.50 (< 0.001)b	25.06 (< 0.001)c	0.00 (0.043)d	2.61 (0.010)e **	7.26 (< 0.001)f **
**Bladder Pathology as assessed by US examination**						
Bladder pathology (as determined by positive global scores from US)	8.42 (0.194)a	5.23 (< 0.001)b	22.95 (< 0.001)c	22.34 (0.057)d **	15.03 (< 0.001)e **	3.98 (< 0.001)f **

^a^p-values comparing proportions reported between Bamako and Koulikoro at baseline in parentheses; ^b ^p-values comparing proportions reported between Koulikoro and Ségou at baseline in parentheses; ^c ^p-values comparing proportions reported between Bamako and Ségou at baseline in parentheses; ^d ^p-values comparing proportions reported between Bamako and Koulikoro at follow-up in parentheses; ^e ^p-values comparing proportions reported between Koulikoro and Ségou at follow-up in parentheses; ^f ^p-values comparing proportions reported between Bamako and Ségou at follow-up in parentheses; ** significant differences at α = 0.05 between baseline and post treatment paired proportions as indicated by McNemar's test

US, ultrasound; PVD, portal vain diameters; PSL, parasternal line; MSL, mid-sternal line; MCL, mid-clavicular line; MAL, mid-axillary line

There were no substantial changes in the prevalence of abnormal liver image patterns from baseline to follow-up in all three areas (Table 4). Only hepatomegaly in Ségou was observed to decrease significantly from baseline to follow-up. Likewise, the prevalence of bladder pathology decreased significantly only in Ségou at follow-up relative to baseline. In the other two areas, follow-up results indicated significant increases relative to baseline. Within both time points, differences in these proportions were statistically significant when comparing Ségou with the other two areas (Table 4).

Results of clinical examinations suggested significant decreases in the prevalence of splenomegaly in Koulikoro and Ségou from baseline to follow-up. The prevalence of splenomegaly in Bamako between the two time points of the study was similar. For the prevalence of hepatomegaly, there was a significant decrease between the two time points of the study only in Ségou whilst in the other two surveyed areas this was similar.

As the descriptive analysis of the results of clinical and US examinations indicated very low prevalences of pathology in Bamako and Koulikoro, the fitting of multinomial models for the changes of pathological manifestations was restricted to the data of children from Ségou. Even within the Ségou area, the fitting algorithm converged only for four outcome variables: liver image patterns, portal hypertension as determined by positive PVD scores from US examination, splenomegaly as assessed by MCL exceeding 2 cm from clinical examination and splenomegaly as assessed by MAL exceeding 2 cm from clinical examination (see Table 5 for results). In addition valid results were obtained only when the schistosome infection status was characterized as simply infected or not infected (see S3 in Additional File 1 for further details).

**Table 5:** Multivariate multinomial logistic regressions for the changes of liver image patterns for Ségou area (n = 427)

Estimates from multinomial model with dependent variable indicating the change of liver image patterns during the 2 time points of the study			
(Adjusted ORs for gender and age, 95% CIs & p-values)			
	Dependent variable: the risk of having pathology at both time points	Dependent variable: the risk of having no pathology at baseline and having pathology at final point of the study	Dependent variable: the risk of having pathology at baseline but cured at final point of the study
Risk factors-Independent variables at baseline(Reference category)			
***Type of schistosomiasis infection related variables at baseline***			
Infected with *S. haematobium *(Not infected with *S. haematobium*)	0.353 (0.080 to 1.560) p = 0.1691	2.339 (0.525 to 10.423) p = 0.2651	0.245 (0.110 to 0.544) p < 0.0011
Infected with *S. mansoni *(Not infected with *S. mansoni*)	34.591 (4.315 to 277.268) p < 0.0011	7.139 (3.513 to 14.506) p < 0.0011	3.081 (1.514 to 6.267) p = 0.0021
**Estimates from model with dependent variable indicating the change of portal hypertension from ultrasound during the 2 time points of the study**			
***Type of schistosomiasis infection related variables at baseline***			
Infected with *S. haematobium *(Not infected with *S. haematobium*)	0.157 (0.036 to 0.695) p = 0.0151	1.541 (0.346 to 6.865) p = 0.5711	0.612 (0.230 to 1.631) p = 0.3271
Infected with *S. mansoni *(Not infected with *S. mansoni*)	2.792 (0.611 to 12.753) p = 0.1851	0.188 (0.043 to 0.813) p = 0.0251	3.076 (1.460 to 6.482) p = 0.0031
**Estimates from model with dependent variable indicating the change of splenomegaly from clinical examination and MCL>2 during the 2 time points of the study**			
***Type of schistosomiasis infection related variables at baseline***			
Infected with *S. haematobium *(Not infected with *S. haematobium*)	0.356 (0.129 to 0.983) p = 0.0461	1.105 (0.136 to 9.009) p = 0.9261	0.482 (0.253 to 0.919) p = 0.0271
Infected with *S. mansoni *(Not infected with *S. mansoni*)	2.946 (1.267 to 6.848) p = 0.0121	0.915 (0.235 to 3.562) p = 0.8991	1.479 (0.900 to 2.432) p = 0.1231
**Estimates from model with dependent variable indicating the change of splenomegaly from clinical examination and MAL>2 during the 2 time points of the study**			
***Type of schistosomiasis infection related variables at baseline***			
Infected with *S. haematobium *(Not infected with *S. haematobium*)	0.505 (0.155 to 1.647) p = 0.2571	1.028 (0.125 to 8.474) p = 0.9791	0.487 (0.256 to 0.929) p = 0.0291
Infected with *S. mansoni *(Not infected with *S. mansoni*)	4.353 (1.681 to 11.272) p = 0.0021	0.953 (0.238 to 3.810) p = 0.9461	1.140 (0.681 to 1.908) p = 0.6191

Note: The comparison group for the dependent variable is 'no pathology at both time points'

ORs, Odds Ratios; 95% CIs, 95% Confidence Intervals

Considering liver image patterns, children with *S. mansoni *infection at baseline compared to those without were more likely to have liver image pathology at one or both time points. In addition, among those children with liver pathology at baseline, those with *S. mansoni *infection at baseline were much more likely than children without to have liver pathology at follow-up (OR for pathology at both time points: 34.6 was much greater than OR for pathology at baseline but no pathology at follow-up: 3.1).

The regression model for portal hypertension from US showed that children with *S. mansoni *infection at baseline would have such pathology at baseline but it did not enable us to predict what is going to happen after treatment (OR for pathology at both time points: 2.8 was similar to the OR for pathology at baseline but not at follow-up: 3.1).

Children with *S. mansoni *infection at baseline compared to those with no *S. mansoni *infection were most likely to have splenomegaly as assessed by clinical examination at both examined time points of interest (OR = 2.9, p = 0.002).

Finally, those children infected with *S. haematobium *at baseline proved significantly less likely than those without *S. haematobium *to have liver pathology at both time points of study (OR = 0.2, p = 0.015; OR = 0.4, p = 0.046 for the risk of portal hypertension and splenomegaly, respectively); those children infected with *S. haematobium *at baseline proved also significantly less likely than those without *S. haematobium *to have liver pathology at baseline and being cured from such morbidity at follow up.

## Discussion

Both *S. mansoni *and *S. haematobium *schistosome species infections are prevalent in much of sub-Saharan Africa and are known to be co-endemic in several countries, such as Mali under study here, with currently available studies contributing useful information with regards to the epidemiology and immunology of each of these separate specific parasitic infections [19-27]. In contrast, however, very little is yet known about potential interactions of concurrent infections, nor as the associated clinical impact of mixed schistosome infections on the human host. Further understanding of polyparasitic interactions is essential in order to guide public health measures in endemic areas [28]. To our knowledge, our study represents the first which uses not only clinical examination but also precise and detailed ultrasonography in order to examine the impact of single versus mixed schistosome species infections on human hosts' liver, spleen and bladder morbidity at the same time, both pre- and post-chemotherapy.

We have demonstrated here that, in general, the risk for baseline liver morbidity amongst these 7-14 year old children, in the form of an abnormal liver image pattern and hepatomegaly, as assessed by positive PSL scores, was lower for co-infections if compared to single *S. mansoni *infections of the same intensity. This finding from Mali agrees with previous results of decreased hepatomegaly, as determined by palpation, in co-infections from Cameroon [6]. Furthermore, our study has also indicated through univariate analysis that at baseline significantly more children with *S. haematobium *only, compared to uninfected children, had liver morbidity (i.e. portal hypertension and hepatomegaly-as assessed by both US and clinical examination) or spleen morbidity as assessed by clinical examination; baseline results from multivariate analysis yielded significant increased risks of hepatomegaly as assessed by positive PSL scores for children with single *S. haematobium *infections compared to uninfected children (ORs for light and heavy intensities of *S. haematobium *infection were estimated respectively as 3.4 and 11.6 in Table 1). Such findings suggest that urinary schistosomiasis alone might also lead to liver and spleen abnormalities, and hence, where possible, potential liver morbidity parameters in *S. haematobium *infections should also be examined during MDA monitoring and evaluation [29]. Indeed, to our knowledge, with the exception of community and hospital surveys performed in Egypt [30,31], there are very few studies that have examined human liver morbidity by US and clinical examinations in *S. haematobium *infection, and our results here may imply that such findings should also be taken into account in the revised assessments of schistosomiasis morbidity and disability adjusted life years (DALYS) [3].

The precise causation of such *S. haematobium*-associated liver and spleen morbidity here remains, however, unclear. One could speculate that children with single *S. haematobium *infections may have had active *S. mansoni *infections in the past, particularly in such co-endemic regions, which could account for the classic *S. mansoni*-associated liver and splenic morbidity observed in *S. haematobium *infected individuals. However, the approximately matched ages of children between infection status groups sampled here may warrant against this as a sufficient potential explanation, although we admittedly cannot fully discount the possibility of some very light current *S. mansoni *infections being missed during the single Kato Katz examinations. An alternative plausible explanation, therefore, may be related to the fact that *S. haematobium *eggs are found in the liver and do cause granulomas, albeit to a lesser extent than *S. mansoni*, as both of these schistosome species involve a hepatic portal system migration phase during their life-cycles [32,33], which could plausibly result in some pathology. In any case, further research into this potentially very important observation, of liver pathology in *S. haematobium *infections (urinary schistosomiasis), and not only *S. mansoni *infections (intestinal schistosomiasis) is warranted.

As regards the bladder morbidity examined here, our results indicated that at baseline children with single and mixed schistosome species infections were significantly more likely to have this risk. Moreover, the risk of having such morbidity was greater in mixed infections than in single *S. haematobium *infections of the same intensity. Once again the mechanisms behind such increased bladder morbidity in mixed as compared to single species infections is uncertain. As we predicted from the results of the Cameroon study in humans [5,6], combined with that know from laboratory mixed species studies in mice [11], synergistic or antagonistic interactions resulting from mixed species infections, and in particular potential interspecific *S. mansoni: S. haematobium *pairings, might be likely to lead to bladder abnormalities as observed by US. In part, mixed species infections within a single host may compete, and increase their individual egg output and subsequent virulence to their host, relative to that produced under single species infections [34]. Indeed, it has been observed in very high infection intensities cases, eggs from either species can be found in a range of 'additional' internal organs [32], although the current analyses controlled for infection intensity and so this alone is unlikely to fully explain the results here. More plausibly the observed results may indicate *S. haematobium *males mating with *S. mansoni *females and the subsequent (infertile) eggs produced from such couplings passing to the urinary oviposition site, thereby reducing the amount of classical *S. mansoni*-induced morbidity whilst increasing the classic *S. haematobium*-associated bladder morbidity. Furthermore, it may be plausible that the infertile, and immunogenically novel, hybrid eggs produced from such female *S. mansoni *and male *S. haematobium *heterologous pairs in the urinary tract may not be as adept at transversing the urinary tract wall, relative to their pure-bred single species counterparts, and hence may more frequently become trapped, and cause granuloma-related morbidity, in the urinary tract and bladder tissues. Whatever the precise aetiology, the results indicate again that any consideration of schistosome-associated morbidity in the human hosts should examine further than the traditional single species 'liver/spleen' for *S. mansoni *and bladder/urinary tract for *S. haematobium.*

When pre- and post-MDA data were analyzed we found that at baseline the highest prevalence of *S. haematobium *infection was observed in Ségou while the highest prevalences of *S. mansoni *infection and co-infections were both found in Koulikoro. At follow-up, prevalences of single and mixed *S. haematobium *infections significantly decreased with the exception of Koulikoro where prevalence of single *S. haematobium *infections significantly increased. Furthermore, the prevalence of *S. mansoni *infection after PZQ treatment significantly increased in all three areas in Mali, as has been reported in Senegal and Egypt [8,9]. Heavy intensities for both *S. haematobium *and *S. mansoni *infections have both significantly decreased in Koulikoro and Segou after PZQ treatment. At follow-up there were significant decreases in liver/spleen and bladder pathologies in the Ségou area while in Bamako and Koulikoro areas there were observed significant increases in bladder pathology. This finding does, as we predicted, highlight the importance of looking at both liver and bladder when assessing morbidity, particularly in areas of mixed foci.

Moreover, multivariate analysis on the two years' data from Ségou area highlighted the causative role of *S. mansoni *infections compared to absence of infection with *S. mansoni *with respect to chronic liver morbidity, both before and after PZQ treatment. More precisely, we found that those children who were infected with *S. mansoni *only compared to those uninfected at baseline were significantly more likely to have liver morbidity at follow-up. For those children who were infected with *S. haematobium *only compared to those uninfected at baseline, they were found significantly less likely to have changes in their liver morbidity during the two years of study. However, intensities of infection were not included in this part of the analysis and further research here is therefore warranted.

We do recognize the inherent limitations of this study, where treatments provided could not be randomized because of ethical reasons associated with a national schistosomiasis control program, in addition to the *a priori *well-known focality of disease in the baseline results of this study and the different number of treatments delivered in each of the three study areas. Other potentially confounding factors such as additional concurrent parasitic diseases, for example malaria, could complicate the interpretation of results, and further research to elucidate these complicating factors is warranted. Nevertheless, we still believe that the results of this study add important insights into polyparasite interactions and their implications to the human host.

## Conclusions

To conclude, this study found decreased liver morbidity in mixed schistosome species infections compared to single *S. mansoni *infections and increased bladder morbidity in mixed schistosome species infections compared to single *S. haematobium *infections. Single *S. haematobium *or *S. mansoni *infections were also associated with liver and spleen morbidity, while only single *S. haematobium *infections were associated with bladder morbidity in these children. PZQ treatment contributed to the regression of some of the forms of such morbidities. The precise biological mechanisms for these observations remain to be fully ascertained but, at least, the estimates of schistosomiasis morbidity DALYs should take the current findings into account.

## Competing interests

The authors declare that they have no competing interests.

## Authors' contributions

AF obtained funding and AF & JPW were the principal investigators. JPW, AF, and AFG participated in the design of data collection. MS, ADK, AL, RD, EBO & AFG participated in data collection. AK (with JPW) drafted the manuscript. AK carried out statistical analysis. All authors contributed to the critical revision of the manuscript for important intellectual content and agreed on submission.

## Pre-publication history

The pre-publication history for this paper can be accessed here:

http://www.biomedcentral.com/1471-2334/10/227/prepub

## Supplementary Material

Additional file 1 **Supplement for the manuscript 'The impact of single versus mixed schistosome species infections on liver, spleen and bladder morbidity within Malian children pre and post Praziquantel treatment'**. This file contains some further statistical analysis that supports the methodology finally used. Click here for file
