# Supplementary material for: The impact of single versus mixed schistosome species infections on liver, spleen and bladder morbidity within Malian children pre- and post-praziquantel treatment
Source: BMC Infect Dis. 2010 Jul 29;10:227. doi: 10.1186/1471-2334-10-227 (PMC2927598; doi:10.1186/1471-2334-10-227)
Supplement: Additional file 1 — Supplement for the manuscript 'The impact of single versus mixed schistosome species infections on liver, spleen and bladder morbidity within Malian children pre and post Praziquantel treatment'. This file contains some further statistical analysis that supports the methodology finally used. [file 1471-2334-10-227-S1.DOC]

**Supplement for the manuscript ‘The impact of single versus mixed schistosome species infections on liver, spleen and bladder morbidity within Malian children pre and post Praziquantel treatment’**

**S.1a**

**WHO classes of intensity for both schistosomiasis species**

|  | **Light-intensity infections** | **Moderate-intensity infections** | **Heavy-intensity infections** |
| --- | --- | --- | --- |
| ***S. mansoni*** | 1-99 epg* | 100-399 epg | ≥ 400 epg |
| ***S. haematobium*** | < 50 eggs/10 ml |  | ≥ 50 eggs/10 ml |

*epg means eggs per gram

**S.1b: Re section 4.1.2**

Below the reviewers can find two tables that contain estimates from multilevel logistic regressions that examine the association between the intensity of schistosomiasis infection and the pathological manifestations at baseline. The difference between these 3 supplement tables (Tables S1, S2 and S3) and Tables 1, 2 and 3 in the actual manuscript is that the estimates presented here derive from models where the intensities of single and dual schistosome species were incorporated as categorical effects instead of linear effects as in the actual manuscript. As it can be seen, trends are similar between the two approaches (i.e. when categorical or linear effects of intensities were fitted) but the problem here is that for some specific categories due to low numbers of children within them, there were estimated very big standard errors and thus very big confidence intervals.

Table S1

| **Estimates from multilevel logistic regressions for liver pathology as assessed by ultrasound** | | | | |
| --- | --- | --- | --- | --- |
|  | **Adjusted OR for gender and age (95 % CI) and p-values for the risk of having abnormal liver image pattern** | | | |
| ***Type of intensity of schistosomiasis infection related variables*** | ***For S.mansoni*** | | | |
| ***For S. haematobium*** | none | light | medium | heavy |
| none | 1 | 2.938  (0.749 to 11.525)  p=0.118 | 8.319  (0.937 to 73.825)  p=0.057 | 18.982  (2.115 to 170.392)  p=0.010 |
| Light | 0.356  (0.121 to 1.043)  p=0.059 | 1.110  (0.333 to 3.699)  p=0.861 | 0.873  (0.182 to 4.184)  p=0.861 | 2.243  (0.576 to 8.729)  p=0.234 |
| heavy | 0.552  (0.148 to 2.060)  p=0.364 | 1.340  (0.326 to 5.515)  p=0.675 | 0.469  (0.071 to 3.079)  p=0.417 | 1.148  (0.253 to 5.202)  p=0.853 |
|  | **Adjusted OR for gender and age (95 % CI) and p-values for the risk of having portal hypertension as assessed by positive PVD scores** | | | |
|  | ***For S.mansoni*** | | | |
| ***For S. haematobium*** | none | light | medium | heavy |
| None | 1 | 1.582  (0.406 to 6.160)  p=0.495 | 1.793  (0.276 to 11.632)  p=0.528 | 0.344  (0.028 to 4.193)  p=0.390 |
| Light | 0.528  (0.187 to 1.493)  p=0.218 | 0.880  (0.247 to 3.129)  p=0.838 | 1.452  (0.412 to 5.111)  p=0.549 | 0.722  (0.198 to 2.637)  p=0.611 |
| Heavy | 1.290  (0.427 to 3.900)  p=0.641 | 1.414  (0.359 to 5.577)  p=0.609 | 0.596  (0.093 to 3.821)  p=0.572 | 0.606  (0.143 to 2.564)  p=0.482 |
|  | **Adjusted OR for gender and age (95 % CI) and p-values for the risk of having of hepatomegaly as assessed by positive PSL scores** | | | |
|  | ***For S.mansoni*** | | | |
| ***For S. haematobium*** | none | light | medium | heavy |
| None | 1 | 18.891  (0.729 to 489.850)  p=0.075 | 0.000*  (NA)  p=0.998 | 152.689  (3.418 to 6820.581)  p=0.011 |
| Light | 3.675  (0.281 to 47.990)  p=0.308 | 0.000  (NA)  p=0.998 | 17.049  (0.532 to 546.864)  p=0.105 | 0.000*  (NA)  p=0.999 |
| Heavy | 12.793  (0.927 to 176.602)  p=0.057 | 19.140  (0.709 to 516.823)  p=0.077 | 0.000*  (NA)  p=0.999 | 0.000*  (NA)  p=0.999 |

*The number of people in these categories and having this specific pathology characteristic was 0 and thus the model did not provide any estimates of ORs there.

**Table S2**

| **Estimates from multilevel logistic regressions** | | | | |
| --- | --- | --- | --- | --- |
|  | **Adjusted OR for gender and age (95 % CI) and p-values for the risk of having hepatomegaly as assessed by MSL >= 2 cm** | | | |
| ***Type of intensity of schistosomiasis infection related variables*** | ***For S.mansoni*** | | | |
| ***For S. haematobium*** | none | light | medium | heavy |
| None | 1 | 2.148  (0.246 to 18.718)  p=0.476 | 3.058  (0.180 to 51.801)  p=0.425 | 8.013  (0.689 to 93.196)  p=0.093 |
| Light | 1.507  (0.317 to 7.164)  p=0.595 | 2.961  (0.509 to 17.224)  p=0.217 | 5.433  (0.901 to 32.770)  p=0.064 | 4.864  (0.816 to 28.997)  p=0.080 |
| Heavy | 2.089  (0.392 to 11.128)  p=0.375 | 1.680  (0.195 to 14.469)  p=0.626 | 1.159  (0.079 to 16.951)  p=0.911 | 4.289  (0.686 to 26.824)  p=0.115 |
|  | **Adjusted OR for gender and age (95 % CI) and p-values for the risk of having hepatomegaly as assessed by MCL >= 2 cm** | | | |
|  | ***For S.mansoni*** | | | |
| ***For S. haematobium*** | none | light | medium | heavy |
| None | 1 | 2.338  (0.270 to 20.214)  p=0.427 | 2.756  (0.157 to 48.410)  p=0.475 | 6.845  (0.599 to 78.281)  p=0.117 |
| Light | 1.453  (0.303 to 6.978)  p=0.629 | 3.325  (0.576 to 19.207)  p=0.172 | 6.589  (1.106 to 39.268)  p=0.039 | 5.073  (0.848 to 30.359)  p=0.074 |
| Heavy | 2.507  (0.461 to 13.629)  p=0.276 | 1.117  (0.084 to 14.826)  p=0.931 | 1.315  (0.090 to 19.277)  p=0.836 | 5.200  (0.811 to 33.348)  p=0.080 |
|  | **Adjusted OR for gender and age (95 % CI) and p-values for the risk of having splenomegaly as assessed by MCL >=2 cm** | | | |
|  | ***For S.mansoni*** | | | |
| ***For S. haematobium*** | none | light | medium | heavy |
| None | 1 | 2.354  (0.994 to 5.574)  p=0.052 | 1.403  (0.422 to 4.667)  p=0.568 | 2.074  (0.580 to 7.414)  p=0.251 |
| Light | 0.900  (0.502 to 1.613)  p=0.713 | 1.156  (0.521 to 2.563)  p=0.712 | 1.253  (0.511 to 3.069)  p=0.610 | 1.205  (0.510 to 2.846)  p=0.661 |
| Heavy | 1.162  (0.562 to 2.402)  p=0.674 | 0.198  (0.038 to 1.022)  p=0.053 | 0.701  (0.199 to 2.466)  p=0.568 | 0.926  (0.329 to 2.608)  p=0.880 |
|  | **Adjusted OR for gender and age (95 % CI) and p-values for the risk of having splenomegaly as assessed by MAL >=2 cm** | | | |
|  | ***For S.mansoni*** | | | |
| ***For S. haematobium*** | none | light | medium | heavy |
| None | 1 | 2.070  (0.829 to 5.172)  p=0.115 | 1.547  (0.429 to 5.572)  p=0.492 | 2.175  (0.560 to 8.441)  p=0.251 |
| Light | 0.907  (0.497 to 1.655)  p=0.741 | 1.118  (0.482 to 2.592)  p=0.789 | 1.263  (0.490 to 3.251)  p=0.618 | 1.160  (0.468 to 2.871)  p=0.741 |
| heavy | 0.876  (0.411 to 1.865)  p=0.722 | 0.376  (0.090 to 1.568)  p=0.172 | 0.654  (0.168 to 2.542)  p=0.527 | 1.075  (0.374 to 3.089)  p=0.890 |

**Table S3**

| **Estimates from multilevel logistic regressions** | | | | |
| --- | --- | --- | --- | --- |
|  | **Adjusted OR for gender and age (95 % CI) and p-values for the risk of having bladder pathology as assessed by positive global scores** | | | |
| ***Type of intensity of schistosomiasis infection related variables*** | ***For S.mansoni*** | | | |
| ***For S. haematobium*** | none | light | medium | heavy |
| None | 1 | 10.320  (2.158 to 49.358)  p=0.005 | 11.938  (1.704 to 83.647)  p=0.014 | 0.145*  (NA)  p=0.746 |
| Light | 8.756  (2.795 to 27.426)  p=0.001 | 12.923  (3.525 to 47.371)  p<0.001 | 24.946  (6.521 to 95.421)  p<0.001 | 11.717  (2.833 to 48.453)  p=0.001 |
| Heavy | 37.390  (11.603 to 120.482)  p<0.001 | 68.286  (18.854 to 247.324)  p<0.001 | 76.508  (18.256 to 320.666)  p<0.001 | 55.902  (14.023 to 222.850)  p<0.001 |

*The number of people in this specific category and having this specific pathology characteristic was 0 and thus the model provided very wide CIs there.

Furthermore, the assumption of linear effects of infection intensity classes was tested using the Akaike information criterion (AIC) to compare models assuming linear effects (with and without an interaction of those linear effects) with models fitting separate effects for each combination of *S. mansoni* and *S. haematobium* intensity classes. When linear effects were incorporated, the intensity classes for *S. haematobium* infection were assigned 0 for ‘not infected’, 1 for ‘light’ and 2 for ‘heavy’ while the intensity classes for *S. mansoni* infection were assigned 0 for ‘not infected’, 1 for ‘light’, 2 for ‘medium’ and 3 for ‘heavy’. The AIC results from these two approaches are displayed below in Table S4.

**Table S4**

| ***What models were used*** | **Intensities of schistosome species as linear effects with interactions** | **Intensities of schistosome species as linear effects without interactions** | **Intensities of schistosome species as categorical effects** |
| --- | --- | --- | --- |
| ***Response variable*** | ***AIC*** | ***AIC*** | ***AIC*** |
| Liver image pattern as assessed by US | 511.1* | 512.0 | 516.4 |
| Portal hypertension as assessed PVD positive scores | 637.5* | 636.3 | 642.2 |
| Hepatomegaly as assessed by positive PSL scores | 173* | NA because the interaction term was significant | 183.0 |
| Hepatomegaly as assessed by MSL >= 2 cm | 431.8* | 430.3 | 443.7 |
| Hepatomegaly as assessed by MCL >= 2 cm | 398.4* | 396.7 | 408.8 |
| Splenomegaly as assessed by MCL >=2 cm | 1188.3* | 1188.6 | 1193.1 |
| Splenomegaly as assessed by MAL >=2 cm | 1101.8* | 1100.6 | 1112.8 |
| Bladder pathology as assessed by positive global scores | 1059.5* | 1058.1 | 1059.1 |

*In all these models the interaction term was not significant

**S.2: Re section 4.2**

Below the reviewers can find two tables that summarize results discussed in the above section.

| **Variables** | **Drop-outs (n=817)** | **Followed-up**  **(n=853)** | **p*** |
| --- | --- | --- | --- |
| Sex-% of males | 47.61 | 51.00 | 0.167 |
| Mean age | 11.25 | 9.62 | <0.001 |
| % infected with *S.haematobium* only | 42.23 | 43.49 | 0.601 |
| % infected with *S.mansoni* only | 6.12 | 6.92 | 0.510 |
| % Co-infected | 25.21 | 28.14 | 0.177 |
| % abnormal liver image patterns as determined by US examination | 3.79 | 6.21 | 0.024 |
| % portal hypertension as determined by positive PVD scores from US examination | 5.75 | 5.28 | 0.669 |
| % of hepatomegaly as determined by positive PSL scores from US examination | 0.61 | 1.41 | 0.106 |
| % of hepatomegaly as determined by MSL>=2cm from clinical examination | 2.82 | 5.04 | 0.020 |
| % of hepatomegaly as determined by MCL>=2cm from clinical examination | 2.57 | 4.45 | 0.037 |
| % of splenomegaly as determined by MCL>=2cm from clinical examination | 10.53 | 16.06 | <0.001 |
| % of splenomegaly as determined by MAL>=2cm from clinical examination | 9.55 | 14.30 | 0.003 |
| % of bladder pathology (as determined by positive global scores from US examination | 10.89 | 15.12 | 0.010 |
| * p values for X^2^ test except for the p-value of mean age which derives from Wilcoxon-Mann Whitney test |  |  |  |

Results when all ages were considered in this analysis

Results when ages of 13 or 14 were excluded from this analysis

| **Variables** | **Drop-outs (n=492)** | **Followed-up**  **(n=812)** | **p*** |
| --- | --- | --- | --- |
| Sex-% of males | 47.97 | 50.99 | 0.291 |
| Mean age | 9.76 | 9.43 | <0.001 |
| % infected with *S.haematobium* only | 42.68 | 43.84 | 0.682 |
| % infected with *S.mansoni* only | 4.88 | 6.53 | 0.221 |
| % Co-infected | 22.15 | 27.46 | 0.033 |
| % abnormal liver image patterns as determined by US examination | 3.86 | 5.91 | 0.104 |
| % portal hypertension as determined by positive PVD scores from US examination | 5.89 | 4.31 | 0.199 |
| % of hepatomegaly as determined by positive PSL scores from US examination | 0.81 | 1.48 | 0.291 |
| % of hepatomegaly as determined by MSL>=2cm from clinical examination | 3.86 | 4.56 | 0.549 |
| % of hepatomegaly as determined by MCL>=2cm from clinical examination | 3.46 | 3.82 | 0.736 |
| % of splenomegaly as determined by MCL>=2cm from clinical examination | 11.59 | 15.27 | 0.062 |
| % of splenomegaly as determined by MAL>=2cm from clinical examination | 10.98 | 13.67 | 0.156 |
| % of bladder pathology (as determined by positive global scores from US examination | 10.77 | 14.78 | 0.039 |
| * p values for X^2^ test except for the p-value of mean age which derives from Wilcoxon-Mann Whitney test |  |  |  |

Comments: By looking through these 2 tables, differences for age between the 2 examined groups still remain significant. The main difference between these two tables is that significant differences in liver pathology outcomes as assessed both by US and clinical examination detected between these 2 groups of children: successfully followed up and dropped out, are not significant any more i.e. when 13 and 14 years old children are excluded from analysis.

However, as mentioned also though in the original manuscript, as the sample size through this approach drops quite dramatically, this creates problems of estimation in both analysis for baseline and evaluation of chemotherapeutic treatment.

**S3: Re section 3.2 and 4.3.3**

Changes of the health characteristics of children between the two time points of the study in relation to their baseline specific schistosomiasis infection status (i.e. infected or not infected) adjusting at the same time for age and sex were analysed using generalized logit multinomial models for all three areas and for each morbidity measure using the PROC LOGISTIC command in SAS version 9.1 (SAS Institute Inc., Cary, NC). We decided to incorporate in these models as covariates the prevalences and not the intensities of single or mixed schistosome species infections based on model fits with prevalence and linear effects of intensity. Table S5 displays these fits based on AIC from these two approaches.

**Table S5**

| ***What models were used*** | **Prevalences of schistosome species** | **Intensities of schistosome species as linear effects** |
| --- | --- | --- |
| ***Response variable*** | ***AIC*** | ***AIC*** |
| Change in liver image pattern as assessed by US at 2 study time points | 590.014 | 590.908 |
| Change in portal hypertension as assessed PVD positive scores at 2 study time points | 482.563 | 494.051 |
| Change in splenomegaly as assessed by MCL>=2 at 2 study time points | 725.634 | 725.741* |
| Change in splenomegaly as assessed by MAL >= 2 cm at 2 study time points | 688.348 | 685.804* |

*SAS software gave a message that the validity of the model fit was questionable when such models were fitted.

In addition, the algorithm converged only when covariates were the continuous variables of age as well as the prevalences of *S. mansoni* and *S. haematobium* infections but without the two latter disentangling for single or mixed schistosome species. When we tried to incorporate the interaction of the prevalences of the two schistosome species and thus get also estimates for ORs referring to co-infections, the algorithms for the corresponding fitted multinomial logistic regression models did not converge.
